# Supplementary material for: Ensemble and single-molecule dynamics of IFT dynein in Caenorhabditis elegans cilia
Source: Nat Commun. 2017 Feb 23;8:14591. doi: 10.1038/ncomms14591 (PMC5331336; doi:10.1038/ncomms14591)
Supplement: Supplementary Information — Supplementary Figures and Supplementary Tables [file ncomms14591-s1.pdf]

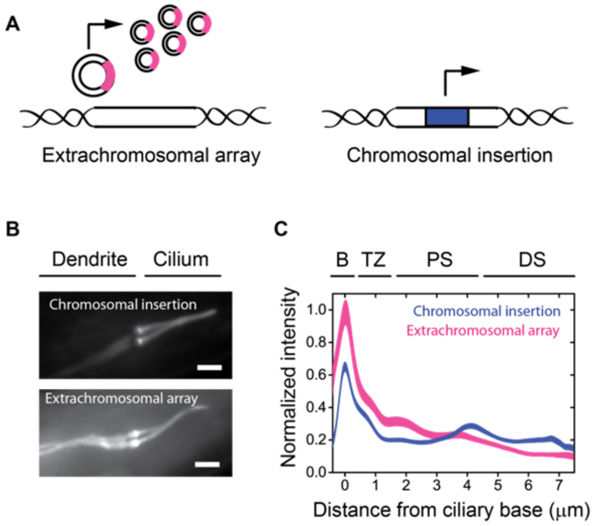

Supplementary Figure 1. XBX-1 expression level affects its distribution in the cilium. (A) Cartoon of two *in vivo* gene expression methods. Transgene injection into the *C. elegans* gonad results in an extrachromosomal array of the gene of interest. Mos-1 mediated single copy insertion (MosSCI) is a chromosomal insertion method. **(B)** Representative summed fluorescence images of XBX-1::EGFP in the dendrite and phasmid cilium (same intensity scaling). Scale bar 2μm. **(C)** Background- and photobleaching-corrected cilium fluorescence intensity in the extrachromosomal array (magenta, n=22 worms) and MosSCI (blue, n=30 worms; same as in Figure 1) constructs. Line thickness represents s.e.m.. B, base; TZ, transition zone; PS, proximal segment; DS, distal segment.

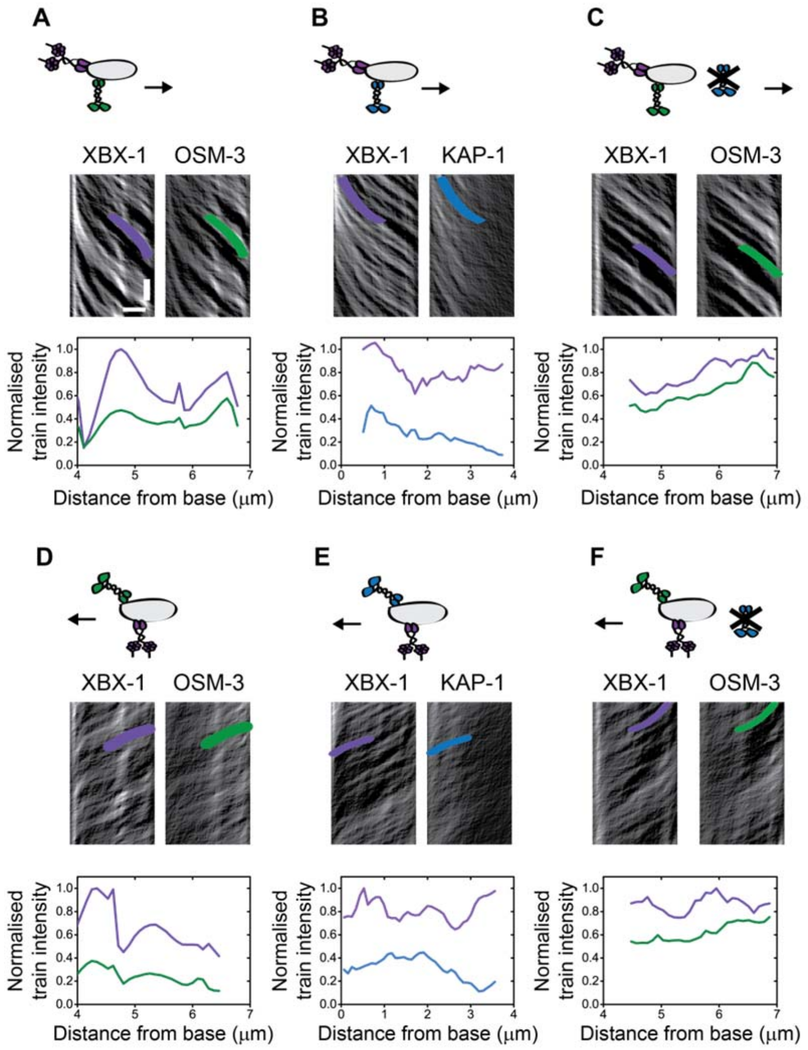

Supplementary Figure 2. Directionality is not modulated by the dynein:kinesin-2 ratio. The dynein::kinesin-2 ratio on trains was obtained from Fourier-filtered kymographs from dual-colour constructs for each direction of movement. A line was drawn along the same train of interest in both channels and corrected for background, photobleaching, bleed-through and excitation intensity. Graphs show example train intensities from Fourier-filtered kymographs for anterograde **(A)** and retrograde **(D)** XBX-1::EGFP (purple) OSM-3::mCherry (green); anterograde **(B)** and retrograde **(E)** XBX-1::EGFP (purple) KAP-1::mCherry (blue) and anterograde **(C)** and retrograde **(F)** XBX-1::EGFP (purple) OSM-3::mCherry (green) without functional kinesin-II. Vertical scale bar 2s, horizontal scale bar 2μm.

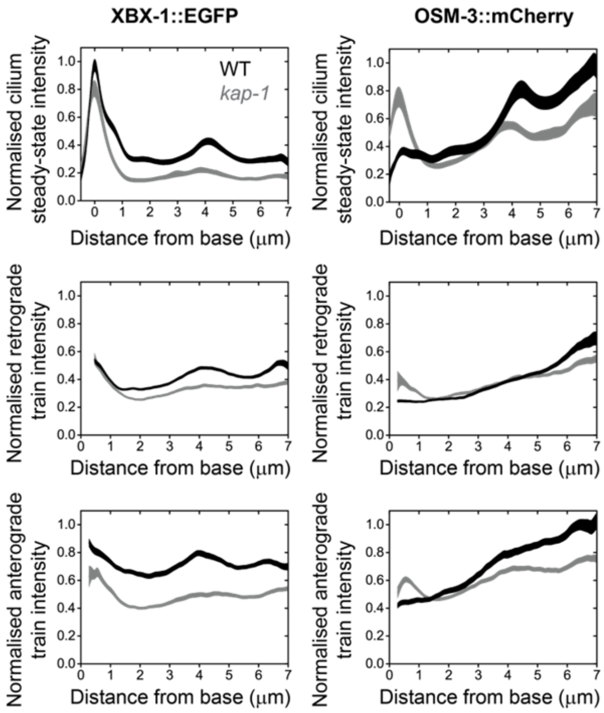

Supplementary Figure 3. Effect of kinesin-II on motor trains and steady-state cilium distribution. XBX-1::EGFP and OSM-3::mCherry normalized fluorescence intensity in double-labelled strains with functional kinesin-II (black; 95 anterograde trains from 24 worms, 86 retrograde trains from 23 worms) and without functional kinesin-II (gray; 56 anterograde trains from 14 worms, 52 retrograde trains from 14 worms). Line thickness shows s.e.m..

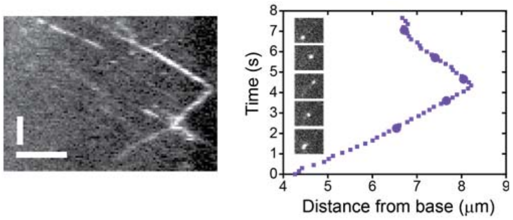

Supplementary Figure 4. XBX-1 tip turnaround. Representative XBX-1 A-to-R directional switch at the tip with corresponding kymograph. Time: vertical; scale bar 2 s. Position: horizontal; scale bar 2 μm.

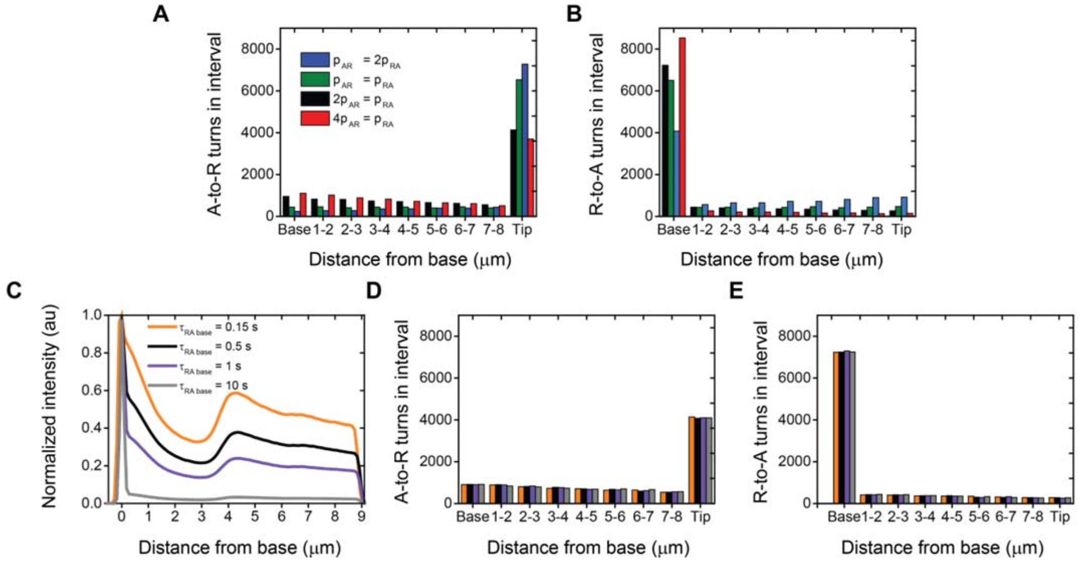

Supplementary Figure 5. Effect of turnaround probability and pause time on IFT dynein distribution in stochastic simulations. (A)-(B) The effect of p_{AR} (probability of IFT dynein A-to-R turn in the cilium) and p_{RA} (probability of IFT-dynein R-to-A turn in the cilium) on IFT dynein turnarounds in stochastic simulations: $p_{AR} = 0.07\ \mu m^{-1}$, $p_{RA} = 0.14\ \mu m^{-1}$ (blue); $p_{AR} = 0.14\ \mu m^{-1}$, $p_{RA} = 0.14\ \mu m^{-1}$ (green); $p_{AR} = 0.14\ \mu m^{-1}$, $p_{RA} = 0.07\ \mu m^{-1}$ (black) and $p_{AR} = 0.28\ \mu m^{-1}$, $p_{RA} = 0.07\ \mu m^{-1}$ (red). **(C)** The effect of $\tau_{RA\ base}$ (IFT dynein pausing in R-to-A turns at the ciliary base) on IFT dynein motor distribution: 0.15s (orange), 0.5s (black), 1s (purple) and 10s (grey). **(D)-(E)** The effect of $\tau_{RA\ base}$ on localizations of A-to-R and R-to-A events in stochastic simulations.

Strain	Genotype	Short notation
EJP206	*vuaSi26 [pJM6; Pxbx-1::xbx-1::EGFP; cb-unc-119(+)] I; xbx-1(ok279) V*	XBX-1::EGFP<br>or Xbx-1
EJP210	*vuaSi210 [pJM10; Pxbx-1::xbx-1::paGFP; cb-unc-119(+)] I; xbx-1(ok279) V*	XBX-1::paGFP<br>or Xbx-1
EJP211	*vuaSi26 [pJM6; Pxbx-1::xbx-1::EGFP; cb-unc-119(+)] I; kap-1(ok676) III; vuaSi1 [pBP20; Pkap-1::kap-1::EGFP; cb-unc-119(+)] IV; xbx-1(ok279) V*	XBX-1::EGFP KAP-1::mCherry<br>or Xbx-1 Kinesin-II
EJP212	*vuaSi26 [pJM6; Pxbx-1::xbx-1::EGFP; cb-unc-119(+)] I; vuaSi2 [pBP22; Posm-3::osm-3::mCherry; cb-unc-119(+)] II; osm-3(p802) IV; xbx-1(ok279) V*	XBX-1::EGFP OSM-3::mCherry<br>or Xbx-1 OSM-3
EJP213	*vuaSi26 [pJM6; Pxbx-1::xbx-1::EGFP; cb-unc-119(+)] I; vuaSi2 [pBP22; Posm-3::osm-3::mCherry; cb-unc-119(+)] II; kap-1(ok676) III; osm-3(p802) IV; xbx-1(ok279) V*	XBX-1::EGFP OSM-3::mCherry<br>*Kinesin-II* or Xbx-1 OSM-3 *Kinesin-II*
EJP217	*vuaSi26 [pJM6; Pxbx-1::xbx-1::EGFP; cb-unc-119(+)]*	Xbx-1 (extrachromosomal array)

Supplementary Table 1. Strains used in this study.

Primer name	Sequence (5’-3’)	Used for
JM3-F	tcgaatcgcaaaaattaatgttcgaATGAGTAAAGGAGAAGAACTTTTCAC	Gibson assembly XBX-1::EGFP and XBX-1::paGFP
JM3-R	GTGAAAAGTTCTTCTCCTTTACTCATtcgaacattaatttttgcgattcgatc	
JM4-F	catggcatggatgaactatacaaaTCTGAATTTATTTGAACTGTGAAATGAATC	
JM4-R	GATTCATTTCACAGTTCAAATAAATTCAGAtttgtatagttcatccatgccatg	
JM7-F	gccttgactagagggtaccGAATATTTATTTGAATTAATTCTTGTTTTTAATT ATTTCG	
JM7-R	CGAAATAATTAAAAACAAGAATTAATTCAAATAAATATTCggtacc cttagtcaaggcctta	
JM8-F	atgcaggtcttccacctacaaAGATCTGCCCACTAGTGAGTCgta	
JM8-R	TACGACTCACTAGTGGGCAGATCTttgtaggtggaagacctgcatttg	
MosI_left	cccaaaatacctccccctcat	MosSCI confirmation
MosI_right	tccggagaaaaacttccaaa	
MosI_int_left	aaattcaggtctgtacggaagc	
pCFJ151_unc199	cgtcagagaggagaggaacg	
JM40-F	ggagacacgaatcgcatatca	Deletion test *xbx-1(ok279)*
JM40-R	ccacgaacggcaattggtta	
JM41-R	tcgaacagagtccaccgttt	

Supplementary Table 2. Primer list.

Strain	M1	M2	M1 control	M2 control
XBX-1::EGFP OSM-3::mCherry	0.92 ± 0.01	0.90 ± 0.01	0.40 ± 0.03	0.44 ± 0.02
XBX-1::EGFP KAP-1::mCherry	0.92 ± 0.01	0.82 ± 0.02	0.43 ± 0.03	0.45 ± 0.03

Supplementary Table 3. Manders’ co-localisation coefficients for XBX-1 with OSM-3 and KAP-1. Coefficients for XBX-1::EGFP OSM-3::mCherry (n=24 kymographs from 24 worms) and XBX-1::EGFP KAP-1::mCherry (n=18 kymographs from 18 worms) are mean ± s.e.m.
